# Supplementary material for: Small sample sizes reduce the replicability of task-based fMRI studies
Source: Commun Biol. 2018 Jun 7;1:62. doi: 10.1038/s42003-018-0073-z (PMC6123695; doi:10.1038/s42003-018-0073-z)
Supplement: Supplementary file 1 — Supplementary Information [file 42003_2018_73_MOESM1_ESM.pdf]

Supplementary Table 1, Results of measurables analyses.

**Original measure**	**Derived measure**	$\mathbf{d_{MAP}}$	$\mathbf{100\times\Delta \mathit{R}^2_{MAP}}$
Mean absolute RMS motion	Mean of mean	-0.17	0.52
	Diff. of mean	-0.06	
	Mean of std. dev.	0.12	
	Diff. of std. dev.	-0.03	
Contrast power	Mean of mean	0.06	0.17
	Diff. of mean	-0.06	
	Mean of std. dev.	0.03	
	Diff. of std. dev.	-0.05	
Hat matrix outliers	Mean of mean	0.00	0.04
	Diff. of mean	-0.08	
	Mean of std. dev.	0.01	
	Diff. of std. dev.	-0.04	
Within-participant variability	Mean of mean	0.14	0.50
	Diff. of mean	-0.11	
	Mean of std. dev.	-0.00	
	Diff. of std. dev.	-0.09	
Between-participant variability	Mean of mean	0.27	0.94
	Diff. of mean	-0.13	
	Mean of std. dev.	0.06	
	Diff. of std. dev.	-0.07	
Sample size	Identity	1.71	31.74

Supplementary Table 2, HCP subject IDs of all subjects included in HCP analyses.

100307	120212	141826	161327	181232	205725	298455	485757	654754	837560
100408	120515	142828	161630	182739	205826	303119	486759	665254	837964
101006	121618	143325	161731	183034	208024	303624	497865	672756	845458
101107	122317	144832	162026	185139	208226	304020	499566	673455	849971
101309	122620	145834	162228	185442	208327	307127	500222	677968	856766
101410	123117	146331	162329	186141	209834	308331	510326	679568	857263
101915	123420	146432	162733	187143	209935	310621	519950	680957	859671
102008	123925	147030	163129	187547	210011	316633	522434	683256	861456
102311	124220	147737	163331	187850	210415	329440	530635	687163	865363
102816	124422	148032	163432	188347	210617	334635	531536	690152	871762
103111	124826	148335	163836	189349	211215	339847	540436	695768	871964
103414	125525	148840	164030	189450	211316	351938	541943	702133	872764
103515	126325	148941	164131	190031	211417	352132	545345	704238	877269
103818	126628	149337	164939	191033	211720	352738	547046	705341	885975
104820	127630	149539	166438	191336	211922	355239	552544	709551	887373
105014	127933	149741	167036	191841	212116	356948	559053	713239	889579
105115	128127	150423	167743	192439	212217	361941	561242	715041	894673
105216	128632	150524	168139	192540	212318	365343	562446	729557	896778
106016	129028	150625	168341	192843	212419	366042	565452	732243	896879
106319	130013	150726	169444	193239	214019	366446	566454	734045	898176
106521	130316	151223	170934	194140	214221	371843	567052	742549	899885
107321	130922	151526	171633	194645	214423	377451	567961	748258	901038
107422	131217	151627	172029	194847	214726	380036	568963	749361	901139
108121	131722	151728	172130	195041	217126	382242	570243	751348	901442
108323	131924	152831	172332	195849	217429	385450	573249	753251	904044
108525	133019	153025	172534	196144	221319	386250	573451	756055	907656
108828	133625	153429	172938	196750	224022	390645	579665	759869	910241
109123	133827	153833	173334	197348	231928	395958	580044	761957	912447
109325	133928	154431	173435	197550	233326	397154	581349	765056	917255
110411	134324	154734	173536	198350	239944	397760	583858	770352	922854
111312	135225	154835	173940	198451	245333	397861	585862	771354	930449
111413	135528	154936	175035	198855	246133	412528	586460	779370	932554
111716	135932	155231	175439	199150	249947	414229	592455	782561	951457

113215	136227	155635	176542	199453	250427	415837	594156	784565	957974
113619	136833	156233	177645	199655	250932	422632	598568	786569	958976
113922	137027	156637	178142	199958	251833	433839	599469	788876	959574
114419	137128	157336	178748	200109	255639	436239	599671	789373	965367
114924	137633	157437	178849	200614	256540	436845	601127	792564	965771
115320	137936	158035	178950	201111	268850	441939	613538	792766	978578
116524	138231	158136	179346	201414	280739	445543	620434	802844	979984
117122	138534	158540	179548	201818	284646	448347	622236	814649	983773
117324	139233	159138	180129	203418	285345	465852	623844	816653	984472
118528	139637	159239	180432	204016	285446	473952	627549	826353	987983
118730	140117	159340	180836	204521	290136	475855	638049	826454	991267
118932	140824	159441	180937	205119	293748	479762	644044	833148	992774
119833	140925	160123	181131	205220	298051	480141	645551	833249	994273
120111	141422	160830							

Supplementary Table 3, HCP tasks and contrasts.

Task abbreviation	Task name	Contrast number	Contrast description
Emot	EMOTION	3	Faces vs shapes
Gamb	GAMBLING	3	Punish vs reward
Lang	LANGUAGE	4	Story vs math
Motor	MOTOR	7	Average motor vs fixation
Rel	RELATIONAL	4	Relation vs match
Social	SOCIAL	6	Theory-of-mind vs random
WM	WM	11	2-back vs 0-back

Supplementary Table 4, Proportions suprathreshold.

Task	Proportion suprathreshold	
	**Liberal**	**Conservative**
3-back	0.115	0.025
ObLoc	0.260	0.131
PPA	0.429	0.227
SST	0.133	0.044
Emot	0.344	0.191
Gamb	0.149	0.040
Lang	0.669	0.498
Motor	0.444	0.273
Rel	0.269	0.116
Social	0.432	0.271
WM	0.408	0.250

Proportion suprathreshold in full-sample analyses for each task, using adaptive thresholds given in Table 1.

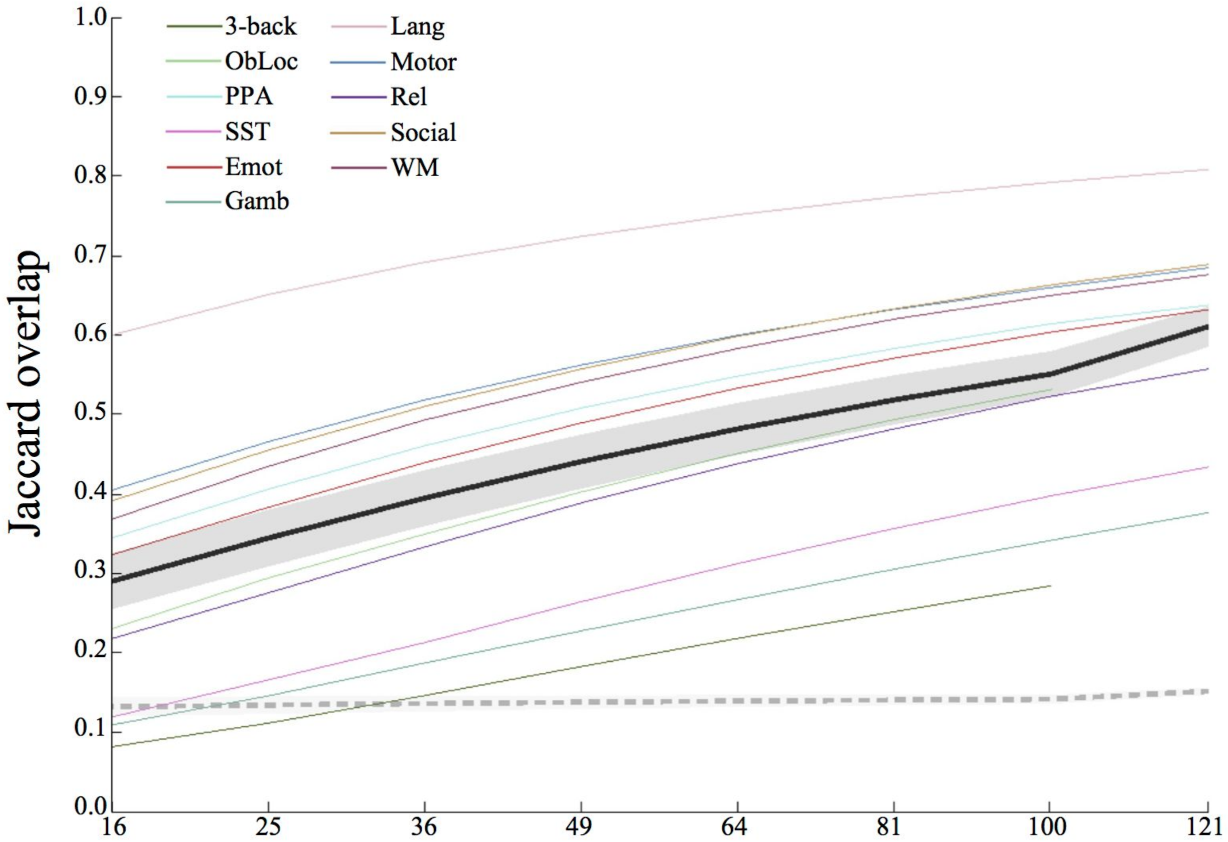

Supplementary Figure 1, Liberally thresholded voxel-level results. Replicability results for voxel-level (thresholded liberally) analyses, measured as the Jaccard overlap between paired group maps—that is the ratio of the number of voxels in the intersection of the two thresholded maps to the number of voxels in the union of the two. Average observed (±1 mean within-task standard deviation) shown in black (dark gray); average null (±1 standard deviation) shown in dashed medium gray (light gray). Also shown are individual task curves for each task (colors given in legend; refer to Table 1 for task abbreviations).

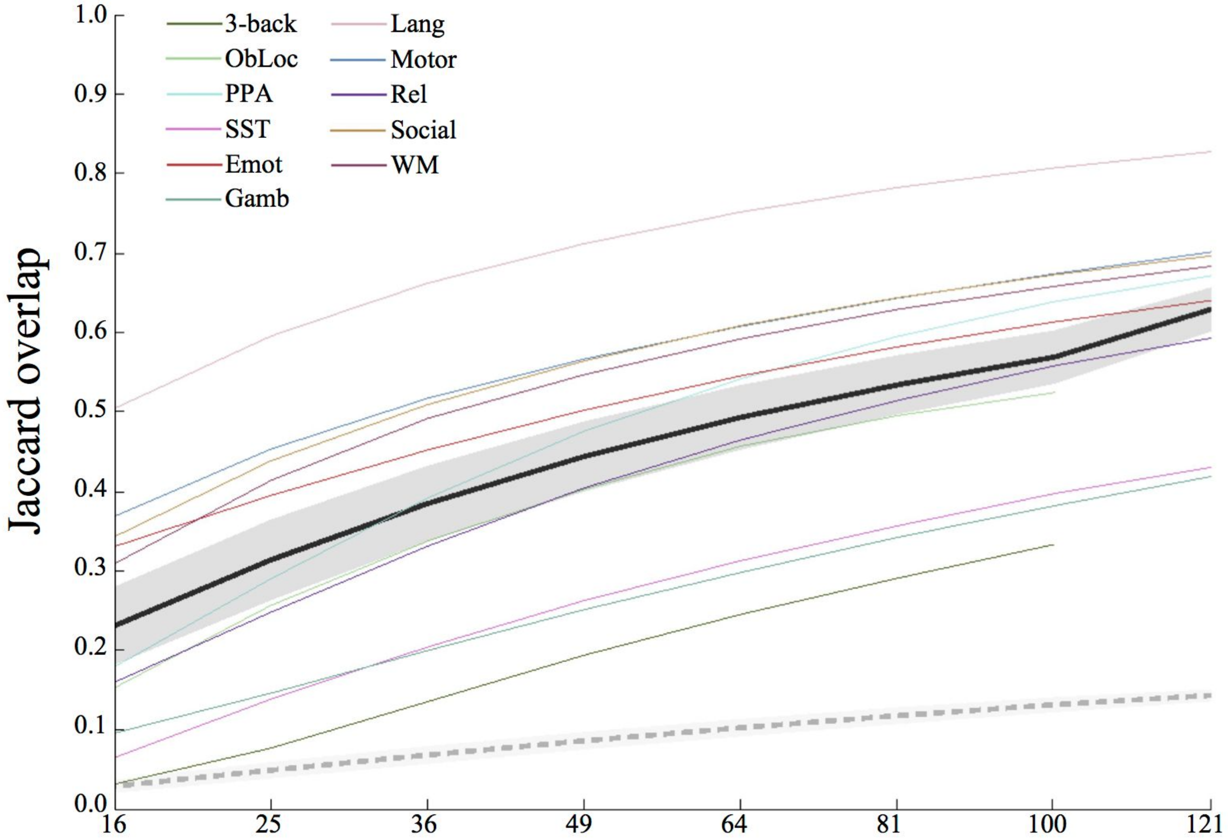

Supplementary Figure 2, Liberally thresholded cluster-level results. Replicability results for cluster-level (thresholded liberally) analyses, measured as the Jaccard overlap between paired group maps—that is the ratio of the number of voxels in the intersection of the two thresholded maps to the number of voxels in the union of the two. Average observed (±1 mean within-task standard deviation) shown in black (dark gray); average null (±1 standard deviation) shown in dashed medium gray (light gray). Also shown are individual task curves for each task (colors given in legend; refer to Table 1 for task abbreviations).

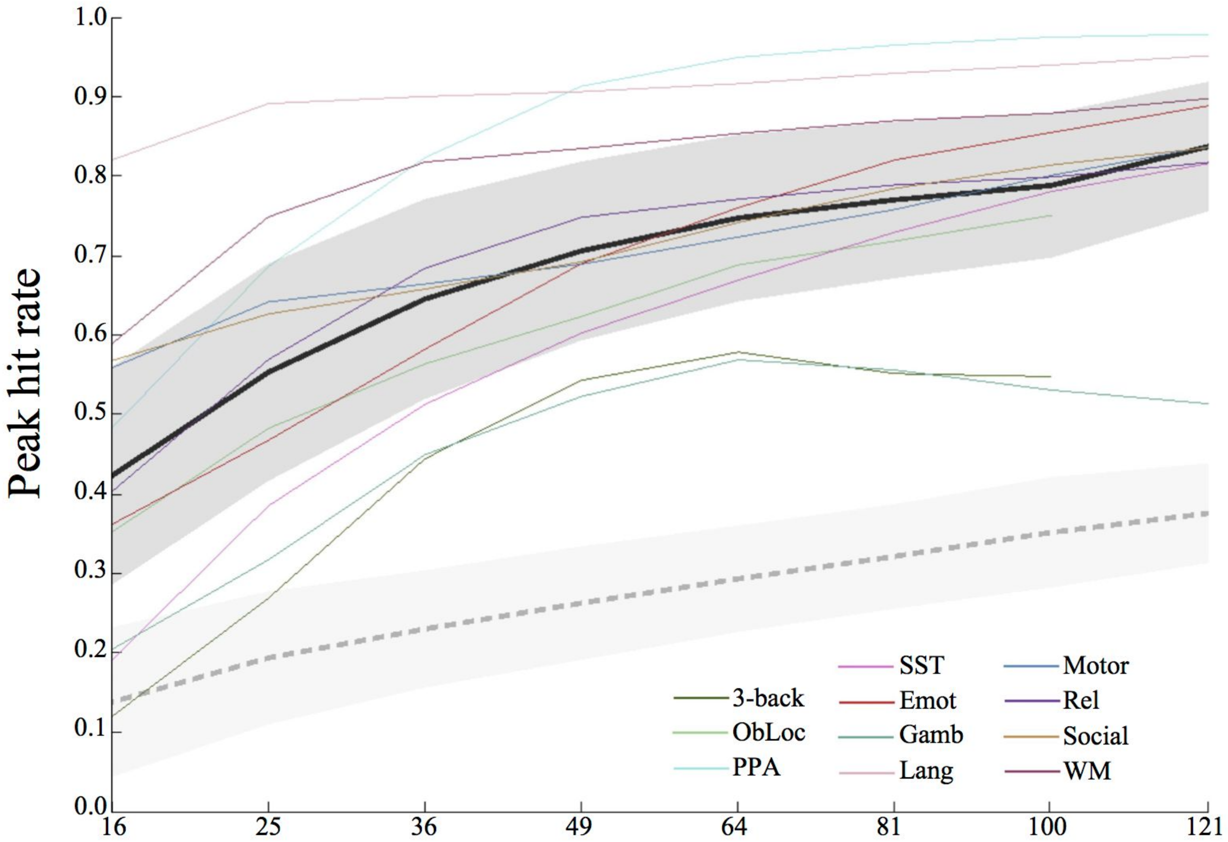

Supplementary Figure 3, Liberally thresholded peak-level results. Replicability results for suprathreshold peak-level (thresholded liberally) analyses, measured as the peak hit rate—that is, the proportion of cluster peaks in one map in each pair that are suprathreshold in the other map. Average observed (±1 mean within-task standard deviation) shown in black (dark gray); average null (±1 standard deviation) shown in dashed medium gray (light gray). Also shown are individual task curves for each task (colors given in legend; refer to Table 1 for task abbreviations).

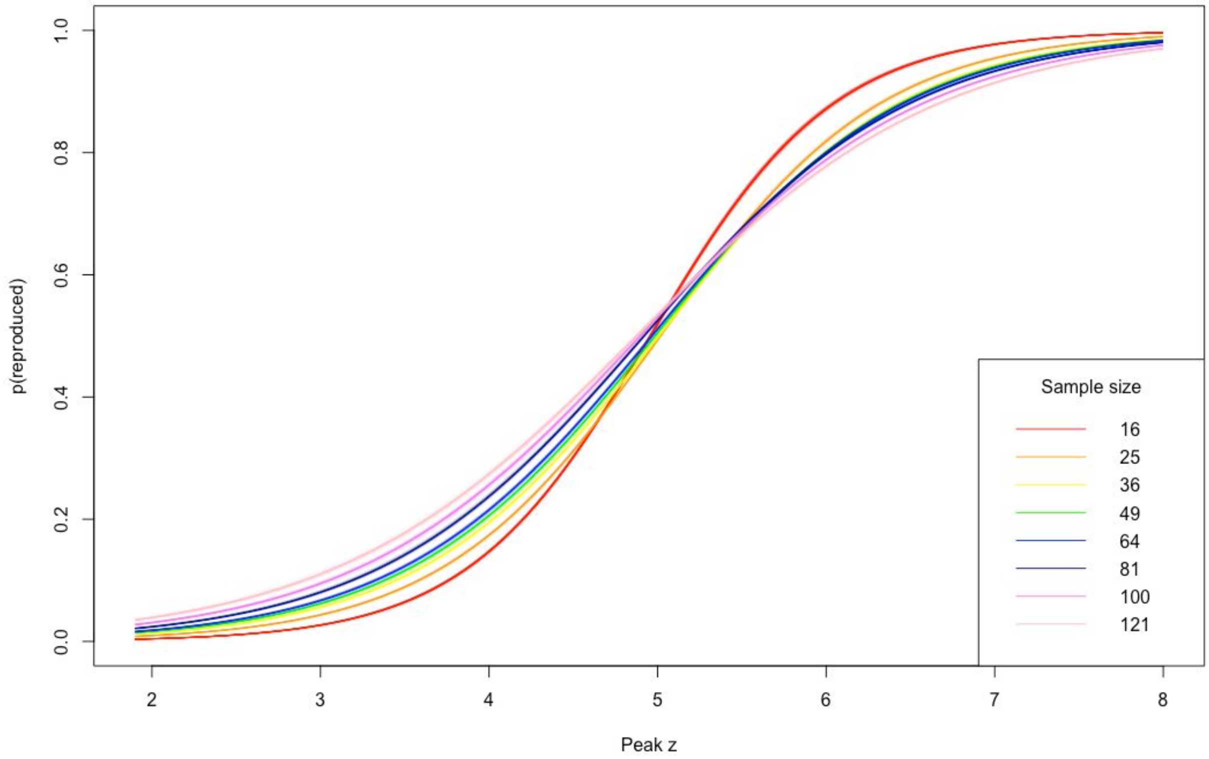

Supplementary Figure 4, Replicability as a function of peak *z* value and sample size (conservative threshold). Curves show logistic regression model-fit results (with 95% confidence intervals) for the relationship between peak *z* value and the probability of the peak being suprathreshold in the corresponding map, separately for each sample size.

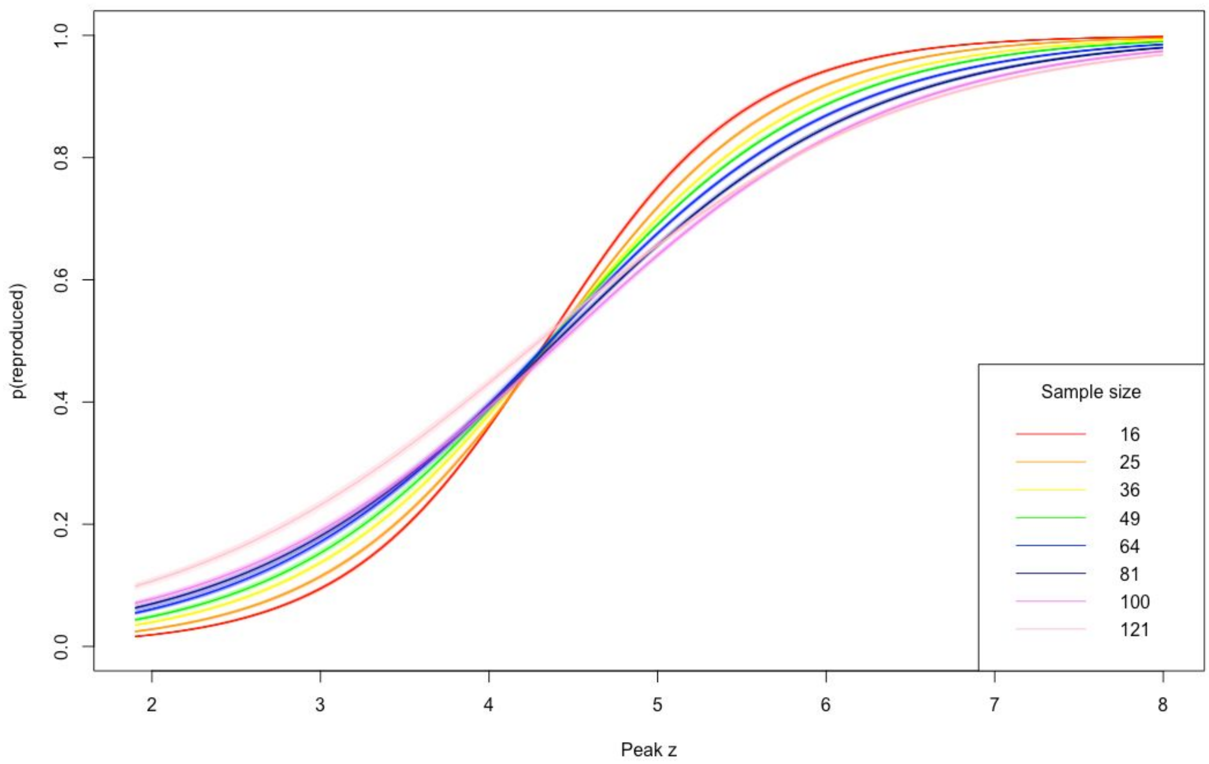

Supplementary Figure 5, Replicability as a function of peak *z* value and sample size (liberal threshold). Curves show logistic regression model-fit results (with 95% confidence intervals) for the relationship between peak *z* value and the probability of the peak being suprathreshold in the corresponding map, separately for each sample size.
